# Transitional cell carcinoma involving graft kidney in a kidney transplant recipient: a case report

**DOI:** 10.1186/s12882-017-0715-2

**Published:** 2017-09-21

**Authors:** Yu Ah Hong, Hyeon Seok Hwang, Hae Joung Sul, Suk Young Kim, Yoon Kyung Chang

**Affiliations:** 1 Department of Internal Medicine, College of Medicine, The Catholic University of Korea, Daejeon St. Mary’s Hospital 64, Daeheung-ro, Jung-gu, Daejeon, 34943 Republic of Korea; 2 Department of Pathology, College of Medicine, The Catholic University of Korea, Daejeon St. Mary’s Hospital, 64, Daeheung-ro, Jung-gu, Daejeon, 34943 Republic of Korea

**Keywords:** Transitional cell carcinoma, Graft kidney, Graft failure, Kidney transplantation

## Abstract

**Background:**

Kidney transplantation (KT) is the treatment option for patients with end stage renal disease (ESRD) to prolong survival and improve quality of life. Although the use of potent immunosuppressive agents increases graft survival in kidney transplantation recipients (KTRs), it may lead to the development of malignancy, including transitional cell carcinoma (TCC). TCC developing in the pelvis of graft kidney is very rare in KTRs.

**Case Presentation:**

A 40-year-old male visited hospital with complaints of nausea, vomiting and gross hematuria. Eleven years ago, he was diagnosed ESRD of unknown origin, and received a living related KT from his father 1 year later. Radiologic findings showed a huge polypoid mass in the pelvis of graft kidney with pelvo-calyceal dilation and a 3.3 cm-sized nodule in aortocaval chain and a 2.5 cm-sized nodule in right iliac chain as TCC stage IV. Sonography-guided percutaneous needle biopsy of pelvis mass in the graft kidney revealed a low grade urothelial cell carcinoma. Radical graft nephroureterectomy was performed and histopathological diagnosis confirmed as a low grade urothelial carcinoma of graft pelvis and ureter lumen, which invaded to perirenal fat and renal parenchyma with lymphovascular presence (T3Nx). The patient started with adjuvant concurrent chemo-radiation therapy and returned to regular hemodialysis.

**Conclusions:**

We report a rare case of TCC in the pelvis of graft kidney with already advanced disease at diagnosis in a young KTR. For the early diagnosis of TCC in KTRs, exposure history to Chinese herb or analgesics should be investigated before KT and high risk population in KTRs should be tightly performed regular postoperative surveillance for TCC and considered of less calcineurin inhibitor-based immunosuppressant protocol.

## Background

Kidney transplantation (KT) is the treatment option for patients with end stage renal disease (ESRD) to prolong survival and improve quality of life. Although the use of potent immunosuppressive agents increases graft survival in kidney transplantation recipients (KTRs), it may lead to the development of malignancy. Especially, the risk for non-melanoma skin cancer, hematological neoplasm, renal cell carcinoma, and thyroid cancer is high after solid organ transplantation [1]. Transitional cell carcinoma (TCC) of urinary tract appears more common in KTRs than in general population, varying its incidence from 0.07 ~ 1.9% [2]. Most of TCC are located in the bladder in KTRs, whereas TCC developing in the pelvis of graft kidney is very rare [3–5]. TCC involving graft kidney can progress decreased graft kidney function and loss the graft in KTRs [6]. The validation of risk factors for early diagnosis of TCC of graft kidney is seriously warranted. Here, we present an uncommon presentation of TCC involving graft kidney in a young KTR with review of literatures.

## Case Presentation

**Fig. 1:**
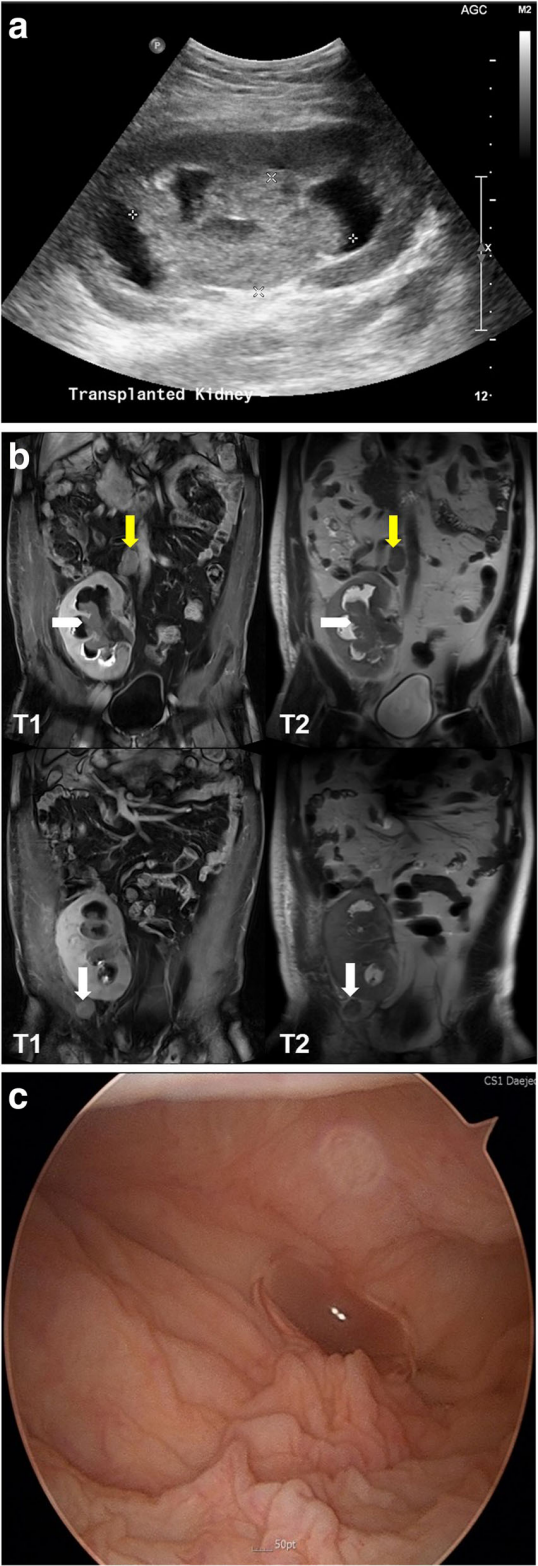
Radiologic examinations and cystoscopic finding in the pelvis mass of graft kidney in a kidney transplantation recipient. **a** Abdominal ultrasonography shows a huge hyperechoic mass (asterisks) in the pelvis of graft kidney. **b** Abdominal magnetic resonance image shows a huge polygonal mass filled within whole pelvis of graft kidney (white arrowhead), a 3.3 cm sized mass in aortocaval chain (yellow arrow) and a 2.5 cm sized nodule in right external iliac chain (white arrow) in coronal view. (Left column: T1 weighted image with gadolinium enhancement, Right column: T2 weighted image) **c** Cystoscopy shows normal graft ureter orifice and normal bladder mucosa

**Fig. 2:**
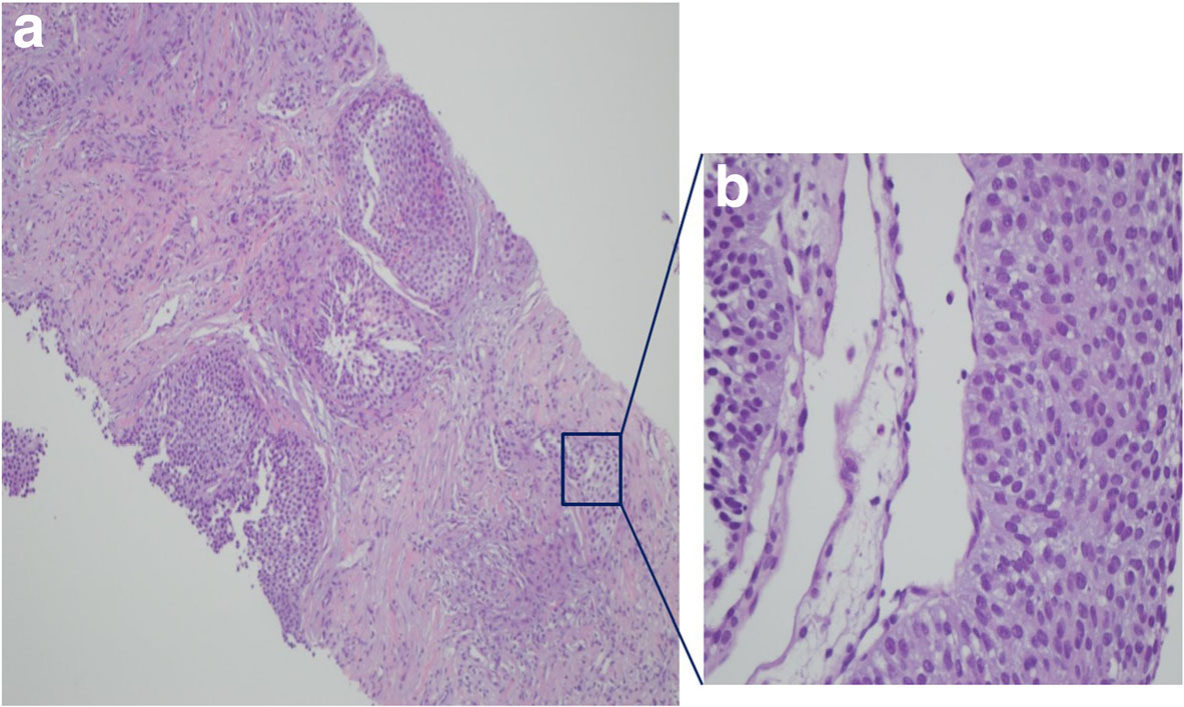
The specimen biopsied by percutaneous needle approach via light microscopy shows low grade urothelial carcinoma (H&E, **a** Original magnification ×40, **b** Original magnification ×400)

**Fig. 3:**
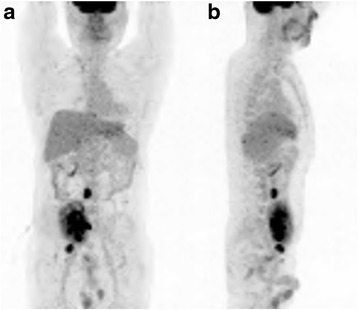
Whole body positron emission tomography (PET) shows a huge mass with high uptake in pelvis of graft kidney, and nodules with high uptake on right external iliac chain and aortocaval chain (**a** Coronal view, **b** Sagittal view)

A 40-year-old male patient visited hospital with complaints of nausea, vomiting and gross hematuria for several days. Eleven years ago, he was diagnosed ESRD of unknown origin, and received a living related kidney transplantation from his father 1 year later. He underwent immunosuppressive agents based on cyclosporine, mycophenolate and corticosteroids. After KT, the patient was performed graft biopsy twice. The 1st biopsy result at 3 months after KT was consistent with acute interstitial nephritis and IgA nephropathy, and 2nd biopsy at 6 years after KT was acute cellular rejection IIb. The graft function had progressively declined to chronic kidney disease stage 4 at the latest visit of the outpatient clinic. At admission, he showed mild fever with body temperature 37.8 °C, and complained mild tenderness on graft kidney. Laboratory data showed white blood cell (WBC) 7400 /mm^3^, hemoglobin 8.2 g/dL, and platelet 258,000 /mm^3^, blood urea nitrogen 87 mg/dL, creatinine 11.2 mg/dL, sodium 141 mEq/L, potassium 5.0 mEq/L, and chloride 105 mEq/L. The urinalysis showed protein (++++), occult blood (+++), red blood cell (RBC) >100 /high power field (HPF), and WBC >100 /HPF. There were not decoy cells in urine cytology, and negative BK virus DNA by urine polymerase chain reaction (PCR). Abdominal sonography demonstrated a 7.9 cm-sized intra-pelvic hyperechoic mass causing obstructive pelvo-calyceal dilation of graft kidney (Fig. 1a). Abdominal magnetic resonance image showed a huge polypoid mass of graft kidney with pelvo-calyceal dilation, and there were a 3.3 cm-sized nodule in aortocaval chain and a 2.5 cm-sized nodule in right external iliac chain (Fig. 1b). There was no invasion to graft ureter orifice and bladder in cystoscopy (Fig. 1c). Whole body positron emission tomography (PET) showed a high uptake mass (standardized uptake value (SUV) max: 14.9) in graft kidney, and reactive lymph nodes with high uptake in right external iliac chain (SUV max: 9.6) and in aortocaval chain (SUV max: 10.1), and then it proved stage IV TCC (cT3N2M0, Fig. 2). Sonography-guided percutaneous needle biopsy of the mass in the pelvis of graft kidney demonstrated a low grade urothelial carcinoma (Fig. 3). Consecutively, radical nephroureterectomy of renal allograft was performed. The post-operative histopathology report confirmed as a low grade urothelial carcinoma of graft pelvis and ureter lumen, which invaded to perirenal fat and renal parenchyma with lymphovascular presence (T3Nx). The patient started with postoperative adjuvant concurrent chemo-radiation therapy and he returned to maintenance hemodialysis. Immunosuppressive agents were discontinued, and no tumor recurrence has been observed during the 24 months after the operation.

## Discussion

TCC in KTRs has different disease characteristics compared with that in general population or dialysis patients. The incidence of TCC after KT has been estimated at around 3 times higher than general population [2, 6]. TCC in KTRs presents as a higher incidence of locally advanced disease or metastasis, and lower age at diagnosis than general population [6]. Recently, Bo Zhang, et al. showed that TCC in KTRs develops more frequently in upper urinary tract than dialysis population [7]. Although TCC can develop at any location in the urinary tract, only few cases have been reported TCC involving graft kidney in KTRs [3–5].

The predisposing factors for TCC in general population are known as old age, male, smoking, chronic exposure to arsenic, chemotherapy such as cyclosphosphamide, radiation, aristolochic acid, Balkan nephropathy, and chronic bladder infections [2, 6, 7]. In KTRs, direct DNA damage by immunosuppressive agents, compromised immune surveillance or viral infection, such as BK virus and human papilloma virus II, may play an important role as possible risk factors of TCC [7, 8]. Interestingly, the incidence of TCC in KTRs in Taiwan and China is relatively high, and the use of aristolochic acid, often presents in some Chinese herbs, has been known as a possible causative factor [9–11]. Previous reports indicated that both exposure to aristolochic acid and immunosuppressive agents in KTRs might work together, and TCC in KTRs would be similar to clinical characteristics of Balkan nephropathy associated TCC. TCC involving graft kidney is very rare phenomena, but could be expected in endemic circumstance that exposed to carcinogen induced DNA damage status [9]. In our case, he had neither history of Chinese herb or analgesics exposure nor BK virus infection after KT. For more definite explanation for the causative factors, diagnostic indices for aristolochic acid or oncogenic DNA mutation after nephroureterectomy might be helpful, but those were not fusible in our patient.

The most common symptoms of TCC in KTRs are painless gross hematuria and pyuria, and other first symptoms of TCC include acute renal failure, hydronephrosis, and chronic urinary tract infection [6–8]. Frequent urinary tract infection in KTRs might delay the diagnosis of TCC [6]. In our case, we confused the cause of intermittent gross hematuria for a while because his primary renal disease diagnosed as 1st graft biopsy was IgA nephropathy. The screening for TCC in KTRs is not yet established and early diagnosis of TCC in graft kidney is very difficult. Although our patient had been performed cancer screening tests annually and checked three times of urine cytology and two times of cystoscopy due to intermittent gross hematuria for 2 years before diagnosis of TCC, early diagnosis of TCC was failed.

The surgical treatment modalities of TCC depend on location and muscle invasion of tumor. In case of TCC in upper urinary tract, radical nephroureterectomy including bladder cuff is a standard treatment, and especially in KTRs, radical resection would be standard of treatment even if patient should return to dialysis [2, 6, 8]. Our patient received radical graft nephroureterectomy, and returned to hemodialysis, and then cisplatin-based adjuvant chemotherapy with radiotherapy was performed 6 times under advanced stage. The prognosis of invasive TCC or TCC in upper urinary tract is poor, and most patients of TCC of graft kidney may return to dialysis [6, 8].

## Conclusions

We report a rare case of TCC of graft kidney in a KTR with already advanced disease at diagnosis. In geographic neighboring location to China and Korean culture familiar to Chinese herb, the incidence of TCC in KTRs might be expected to increase. Although we did not elucidate causative risk factors of TCC in our patient, the investigation of exposure history to Chinese herb or analgesics is important to early detect TCC in KTRs. High risk population in KTRs should be tightly performed regular postoperative surveillance for TCC and considered of less calcineurin inhibitor-based immunosuppressant protocol.

## Abbreviation

ESRD: End stage renal disease
KT: Kidney transplantation
KTR: Kidney transplant recipient
PET: Positron emission tomography
SUV: Standardized uptake value
TCC: Transitional cell carcinoma

## References

[1] KDIGO clinical practice guideline for the care of kidney transplant recipients. Am J Transplant. 2009;9 (Suppl 3):S1–155. 10.1111/j.1600-6143.2009.02834.x 19845597

[2] Hevia V, Gomez V, Diez Nicolas V, Alvarez S, Gomez Del Canizo C, Galeano C (2014). Development of urologic de novo malignancies after renal transplantation. Transplant Proc.

[3] Hevia V, Gomez V, Alvarez S, Diez Nicolas V, Gomez Del Canizo C, Orosa A (2013). Transitional cell carcinoma of the kidney graft: an extremely uncommon presentation of tumor in renal transplant recipients. Case Rep Transplant.

[4] Mokos I, Pasini J, Stern-Padovan R, Mrsic S, Ries S (2006). Conservative surgical treatment of low-grade urothelial carcinoma in the renal allograft recipient: a case report. Transplant Proc.

[5] Takaoka E, Miyazaki J, Kimura T, Kojima T, Kawai K, Murata Y (2014). Concurrent urothelial carcinoma in the renal pelvis of an allograft kidney and native recipient bladder: evidence of donor origin. Jpn J Clin Oncol.

[6] Cox J, Colli JL (2011). Urothelial cancers after renal transplantation. Int Urol Nephrol.

[7] Zhang B, Shen C, Han WK, Yu W (2014). Comparison of clinicopathologic characteristics of urothelial carcinoma between patients after renal transplantation and on dialysis. Transplantation.

[8] Salvatore SP, Myers-Gurevitch PM, Chu S, Robinson BD, Dadhania D, Seshan SV (2016). Polyoma (BK) virus associated urothelial carcinoma originating within a renal allograft five years following resolution of polyoma virus nephropathy. Clin Nephrol.

[9] Grollman AP, Jelakovic B (2007). Role of environmental toxins in endemic (Balkan) nephropathy. October 2006, Zagreb, Croatia. J Am Soc Nephrol.

[10] Li XB, Xing NZ, Wang Y, Hu XP, Yin H, Zhang XD (2008). Transitional cell carcinoma in renal transplant recipients: a single center experience. Int J Urol.

[11] Wu MJ, Lian JD, Yang CR, Cheng CH, Chen CH, Lee WC (2004). High cumulative incidence of urinary tract transitional cell carcinoma after kidney transplantation in Taiwan. Am J Kidney Dis.

